# First in-human intervention using a semi-automated robot for tooth restorative treatment

**DOI:** 10.21203/rs.3.rs-6455412/v1

**Published:** 2025-06-03

**Authors:** Christopher Ciriello, German Gallucci, Phillip Getto, Joseph Doeringer, Jacob Rosen, Kevser Pala

**Affiliations:** Perceptive Technologies; Harvard School of Dental Medicine, Harvard University; Perceptive Technologies; Perceptive Technologies; Perceptive Technologies; Harvard School of Dental Medicine, Harvard University

**Keywords:** robotics, robotic dentistry, robot-enhanced procedures, restorative dentistry, dental caries, digital technology, health services accessibility

## Abstract

The advent of digital technologies has not only disrupted but also revolutionized dentistry by enhancing precision, efficiency, and predictability ^1,2^. Robotic technologies represent the next transformative leap, by enabling automated workflows that minimize human error and streamline treatments ^3,4^. In restorative dentistry, traditional crown preparation involves manual shaping of the tooth, obtaining impressions, and multiple patient appointments. In this study we present data from the first in-human study performed by a semi-automated robotic tooth preparation system (SARP). SARP digitally preplans the tooth preparation, executes it with sub-50μm precision, and allows the pre-manufacturing of restorations for same-day delivery. Among the six patients who completed the procedure, no adverse events occurred. The root mean square deviation of the final preparation relative to the planned shape was 39μm. Prepared crowns (n = 5) achieved a good-to-excellent fit and were permanently cemented during the same visit. All participants reported no pain during or after the procedure using SARP. These findings suggest that SARP can enhance procedural precision, reduce treatment times, and improve patient satisfaction while increasing practice efficiencies. A future integration of SARP with advanced imaging modalities, (i.e. optical coherence tomography), is expected to further improve treatment options ^5–8^. Larger, controlled trials are currently planned to validate these results, assess long-term outcomes, and explore the system’s potential to improve cost-effectiveness and expand access to restorative dental care.

## Introduction

Dental caries, commonly known as tooth decay, remains one of the most prevalent health conditions globally, affecting individuals of all ages ^9–12^. Left untreated, caries can result in structural tooth damage, chronic pain, tooth loss, and their effects on systemic inflammatory responses are also being investigated ^13,14^. Research has linked untreated caries to hyperglycemia ^15^, type II diabetes, metabolic dysfunction ^16^, vascular inflammation ^17^, arterial hypertension ^18^, and respiratory infections that could lead to development of conditions such as asthma ^19^,^20^. These far-reaching health impacts emphasize the need for accessible, efficient, and effective treatments.

The advent of digital technologies has fundamentally transformed dentistry, revolutionizing workflows and expanding treatment possibilities ^1^. These innovations have made dental interventions more predictable and comprehensive, with faster progress and enhanced treatment planning. Computer-assisted design (CAD) and computer-aided manufacturing (CAM) have significantly improved efficiency of dental procedures, including clinical treatments and manufacturing procedures ^2,21^. Building on this digital evolution, the incorporation of robotic technologies represents the next disruptive leap in dental care. Robotic and navigational systems, already explored in both medicine and dentistry, have demonstrated their potential to enhance procedural precision and minimize invasiveness ^3,4^.

The use of robotic systems in dentistry aims to increase treatment accuracy, treatment success and improve patient experience ^22^. Robotic devices offer the capability to execute dental procedures with minimal human involvement, reducing susceptibility to human errors while improving consistency and predictability. The capability of current robotic devices to fulfill these expectations in dentistry is not conclusive and is currently being investigated ^23^. This automation of the dental procedures could shorten treatment times and reduce the number of required patient appointments. Traditionally in restorative dentistry, the process involves manually preparing a tooth, taking an impression, and fabricating a crown, that is then delivered during a separate appointment. With robotics, this workflow is transformed: tooth preparation is preplanned digitally, executed by a robot with high precision, and based on the digital simulation of the preparation a crown is prefabricated and delivered during the same visit as the tooth preparation. This advancement would not only increase precision and streamline workflows, but may also significantly enhance patient satisfaction, improve the practice’s efficiency, and expand access to dental care. Thus, streamlining these processes could make dental treatments more accessible to underserved populations, addressing both logistical and financial barriers.

**Figure 1:**
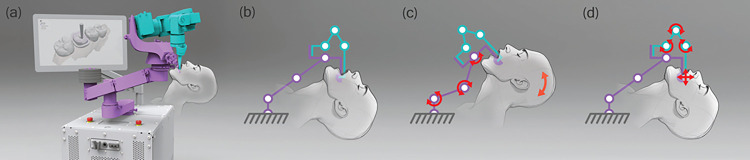
Illustration of the robotic semi-automated robotic preparation system (SARP) (Perceptive Technologies); A) SARP device with patient illustration; B) robotic arm of the SARP device attached to the target region of the patient’s mouth; C) movements of the support arm joints in response to patient movement; D) movement of the robot arm to accomplish the treatment.

**Figure 2:**
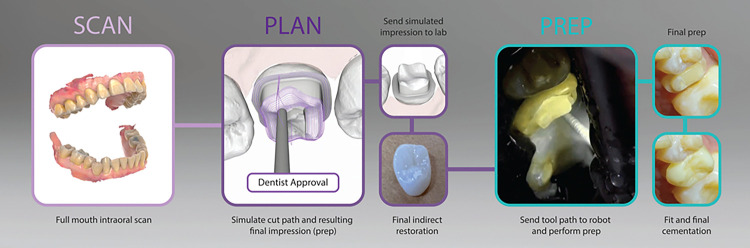
Workflow overview. First steps include digital impression, followed by the digital preparation plan. After receiving the dentist’s approval, the final restoration is prefabricated according to the simulated final impression of the digitally prepared tooth. Last three images show the actual robotic tooth preparation, the final preparation and cemented crown.

In this study, we present an early feasibility clinical intervention of a semi-automated robotic preparation system (SARP) (Perceptive Technologies, Boston, MA. USA) capable of preparing teeth for crowns with sub-50μm accuracy (Fig. 1). By simulating the final preparation in advance and executing the procedure, SARP aims to standardize tooth preparation, minimize unnecessary removal of healthy tooth structures, and enable the fabrication of restorations pre-operatively for immediate placement (Fig. 2, Video 1). This innovative approach dramatically shortens procedural times and reduces the number of patient visits required, including those for lab manufactured crowns and not just CAD/CAM crowns, improving patient experience and increasing efficiency within the dental practices.

Robotic tooth preparation has shown promise in *in-vitro* studies ^24–30^. However, clinical studies testing robotic tooth preparation on human subjects are lacking. Additionally, previous research has highlighted the need for systems capable of accommodating patient movements in real clinical scenarios, an essential factor for real-world implementation.

This initial single group feasibility study evaluates the safety and accuracy of SARP in tooth preparation and immediate crown fitting. The outcomes from seven enrolled participants, six of whom completed the procedure, provide the preliminary data that lay the foundation for future large-scale clinical trials. These planned trials will further assess the system’s safety, efficacy, cost-effectiveness, and the potential to expand access to high-quality restorative care for patients worldwide.

## Results

### Study population and procedure feasibility

Seven participants meeting the inclusion criteria were enrolled. One participant was withdrawn due to the inability to fit the tooth clamp properly without contacting the patient’s cheek. Six participants underwent tooth preparation using the SARP device. Five male and one female participant were included (aged 18–60).

### Safety

No adverse events were observed or reported during the SARP procedure.

### Pre-operative training

Dentist spent approximately 3 hours training along with staff prior to the first procedure. This training involved acting out a simulated procedure on a manikin with support staff from Perceptive onsite to assist, followed by a simulated procedure by the doctor on his/her own. Whenever the doctor or staff required further support, Perceptive was onsite to assist to provide help and answer questions. No additional training was required after the initial patient.

### Patient-reported outcomes

Patients scored the tooth, gingiva, jaw and head discomfort experienced during the procedure using the Wong-Baker Pain Rating Scale. All participants reported no discomfort during or after the procedure, aligning with the primary safety objectives and the patients reported that the semi-automated approach reduced discomfort compared to conventional procedures they had previously experienced. All patients indicated that they felt better being subjected to the SARP than with comparable dental procedures conducted by a dentist. Most subjects (6/7) indicated they were relaxed (none of the patients reported any form of anxiety) from seeing the robot moving in close proximity to their face. One subject indicated that they felt apprehensive at first, but that apprehension quickly dissipated as the procedure began.

### Robotic tooth preparation, preparation accuracy and crown fit

**Figure 3:**
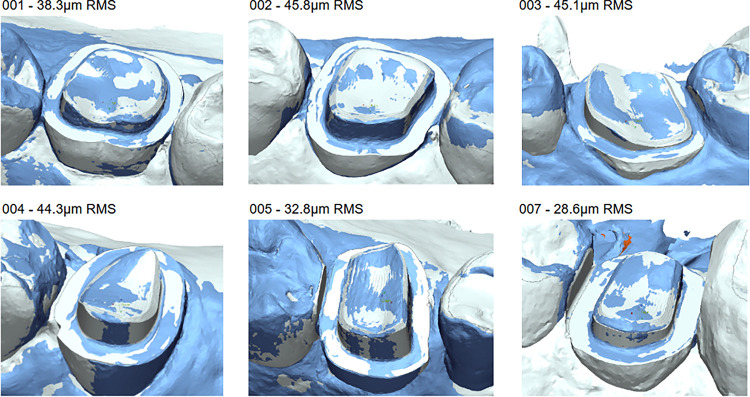
Superimposed intraoral scans showing planned preparation (white) versus actual preparation (blue) for each subject to complete the study. The actual preparation scan was aligned to the planned preparation model using the margin, axial wall and occlusal surfaces. The RMS accuracy of the actual preparation shape is listed beside each subject number.

In all cases, the dentist connected the SARP device to the patients’ target tooth and adjacent teeth area using the prefabricated customized tooth clamp. After activation, the SARP device executed the dentist approved preparation on the target tooth, while the dentist only observed the procedure, until the robotic tooth preparation was completed. During these initial procedures, the dentist maintained their foot on the rheostat. The rheostat acted as a “Deadman’s Switch”, controlling both the activation of the highspeed handpiece and the movement of the robot along the trajectory. The mean root mean square (RMS) (±SD) deviation between the planned and achieved tooth preparation shapes was 39.22 μm (± 7.27) (Figure 3). The shape analysis for the prepared teeth when overlaid onto the planned tooth shape using a best-fit alignment based solely on the prepared surfaces demonstrated an RMS deviation in all cases of less than 50 μm (range 28.6 – 45.8 μm; Table 1). When the prepared tooth scan was aligned to the planned shape using best-fit alignment of the adjacent tooth occlusal surfaces, thereby assessing both the preparation’s location and shape, the RMS deviation was less than 110μm (range 68.5 – 109.7 μm; Table 1). Adjacent teeth were examined for iatrogenic damage: in five cases there was no damage visible to the unaided eye, and one case had minor damage as anticipatedin the pre-approved digital preparation plan as the predesigned preparation plan required a minimal interproximal reduction which was approved by the dentist (Table 1)

Five out of six crowns were rated as having good to perfect fit (scores of 4–5) and were permanently cemented at the time of the procedure. One crown showed partial fit at the margins and occlusal contacts and served as a temporary restoration. A post-study analysis attributed this imperfect fit to a manufacturing variation rather than a preparation error, suggesting that a deviation had occurred during the manufacturing of the restoration itself.

## Discussion

This early feasibility study demonstrated that semi-automated robotic tooth preparation is achievable with no dental observed adverse events in the procedure and no adverse reactions by the patients, sub-50μm accuracy, and immediate crown placement. These outcomes, while limited by the small sample size, suggest that a semi-automated approach could streamline the restorative workflow, reduce patient chair time, and potentially improve outcomes by minimizing unnecessary tooth structure removal.

One of the most significant advancements of SARP lies in its ability to achieve sub-50μm accuracy despite natural patient movements. Traditionally, static positioning or continuous tracking of the patient’s head motion has presented a major challenge. In contrast, SARP is designed to “move with” the patient. It accomplishes this by using a per-patient customized tooth clamp that rigidly couples the robotic system to the patient’s own dentition, analogous to a clinician’s finger rest during manual tooth preparation. The robot’s weight is supported by a specialized suspension system, allowing it to respond passively to minor patient movements without losing its precise orientation relative to the tooth. This simple, yet effective design eliminates the need for continuous patient-tracking imaging or complex motion compensation algorithms, ensuring that the cutting path remains stable and accurate even when the patient is awake and able to make minor movements (Fig. 1). As a result, SARP maintains sub-50μm precision throughout the procedure, a level of accuracy that supports the pre-operative fabrication and immediate placement of restorations.

The ability to pre-manufacture restorations based on a planned final impression and then the execution of the preparation to match that procedural plan is a significant dental advancement. This approach reduces procedure time and the need for multiple appointments, thereby lowering costs and improving convenience for the patients. Furthermore, automating part of the procedure would enhance access to care in regions with limited dental professionals, as standardized protocols and reduced patient chair time would facilitate treating more patients with limited existing staff resources by improving workforce productivity.

However, these findings represent only an initial step. Larger-scale clinical trials with longer follow-up periods, and more complex clinical scenarios are needed to assess durability, long-term outcomes, and cost-effectiveness of the procedure. Moreover, while this study focused on conventional crown preparations, future studies should explore minimally invasive preparation designs to further preserve healthy tooth structure. Integration with advanced imaging modalities, such as optical coherence tomography (OCT), could further enable automated caries detection and selective removal of diseased tissue, further allowing for more improved outcomes ^5–8^.

Finally, widespread clinical adoption will hinge on reducing the cost and complexity of the system.

## Conclusions

The initial clinical experience with SARP suggests that semi-automated robotic dentistry can be performed safely and with high accuracy. Although this study had a limited enrolment, confirmation in a larger and more diverse population is needed, the results highlight the potential of robotic dental systems to enhance precision, efficiency, and patient experience in restorative dentistry. Future research will aim to refine the technology, reduce tooth structure removal, incorporate advanced imaging, and evaluate the system’s impact on broader clinical outcomes and healthcare delivery.

## Materials and Methods

### Ethics and study design

This investigation was conducted as an Early Feasibility Study (EFS) with a single group at a single clinical site. The study followed the ethical principles outlined in the Declaration of Helsinki and adhered to FDA Good Clinical Practice Regulations. The study protocol (Protocol CP-00003) was reviewed and approved by the local ethics committee (Comité de ética en investigación C.F.C. S.A.S, Barranquilla, Colombia). All participants provided written, informed consent prior to enrollment.

### Study population

Participants were recruited at a dental clinic, Clinic Carlos Fernandez de Castro Advanced Dentistry, Barranquilla, Colombia. Patients that required a single tooth-supported restoration in the posterior region of the maxilla (upper maxillary second bicuspid, first molar, or second molar; [i.e., teeth 2, 3, 4, 13, 14, or 15]) were considered eligible for the study.

### Inclusion criteria

- Adult (≥ 18 years old) patients willing to participate and provide informed consent.
- Patients indicated for a single-unit tooth-supported crown restorative treatment in the specified upper maxillary teeth.
- Sufficient number of intact teeth to ensure proper clamp attachment (at least 3 teeth to be attached within the clamp) for the robot.
- Adequate mouth (opening (Class I equivalent, inter-incisal distance ≥ 45 mm) and ability to remain fully supine for 45 min and maintain an open mouth for up to 20 min)

### Exclusion criteria

- Pregnancy.
- Existing major dental work in the applicable quadrant (crowns, bridges, veneers, inlays, onlays, or implants).
- Moderate to severe periodontal disease (Stage 3 or greater).
- Structurally unsound teeth or untreated caries in any teeth to be attached to the clamp.
- Dental crowding of the target tooth (adjacent tooth overlap).
- Target tooth lacking adjacent teeth (except one adjacent tooth).
- Target tooth serving as an abutment for fixed or removable prostheses.
- Inability to tolerate dental apparatus for prolonged periods.
- Target tooth incompatibility with the clamp.
- Underlying neck/spinal mobility issues, temporomandibular joint disorders, epilepsy, bipolar disorder, schizophrenia, substance abuse history, cognitive disorders, unrestored tooth fractures, existing amalgam restorations in the target tooth, or need for a crown related to implant procedures, malocclusion correction, or GERD-related erosion.
- High dental anxiety (Corah’s Dental Anxiety Scale ≥ 9)
- Known allergies or adverse reactions to local anesthetics.

### Pre-enrollment preparation

If the target tooth required basic restorative care (e.g., caries treatment, build-up) to achieve structural soundness, it was performed prior to enrollment under standard clinical care protocols.

### Intervention (device and procedures)

This study evaluated a semi-automated robotic preparation system designed to prepare teeth for crowns according to a preoperative digital plan. The workflow used for the semi-automated robotic tooth preparation is illustrated in Figure 2. The system included a custom tooth clamp, and a robotic arm equipped with a dental handpiece.

### Clinical procedures

Participants attended three visits:

#### 1. Visit 1 (exam, screening, digital scan):

- Eligibility was confirmed, and digital impressions (STL files) of the target tooth and adjacent teeth were obtained using an intraoral scanner.
- The digital impression was used to create a simulated final preparation plan, which was reviewed and approved by the investigator.

#### 2. Between visits:

- A 3D-printed clamp insert was manufactured for each patient, matching their dentition for stable robot-to-tooth registration.
- The final simulated preparation served as a guide for fabricating a chairside zirconia restoration in advance of the treatment visit.

#### 3. Visit 2 (tooth preparation and crown placement):

- Local anesthesia was administered.
- The custom tooth clamp, with its 3D-printed insert, was placed intraorally and secured. The robotic system was attached to the clamp’s base.
- A registration scan of the target tooth, adjacent teeth, and the operating field was performed. This data, combined with robot joint angles, aligned the planned preparation path with the patient’s anatomy.
- Collision testing was conducted via simulation software to ensure no interference among the handpiece, clamp, or opposing dentition.
- The dentist activated the robotic preparation using a foot pedal (dead man’s switch) for safety. The robot prepared the tooth according to the digital plan.
- After preparation, a final scan documented the outcome. The pre-manufactured crown was placed, and its fit assessed and adjusted as needed before permanent cementation.
- Participants completed a brief postoperative questionnaire.

#### 4. Visit 3 (postoperative assessment):

- Participants returned approximately two weeks later for a postoperative evaluation, ensuring the stability and success of the restoration.

### Outcome Measures

#### Primary outcome (safety)

Safety was defined as the absence of serious adverse events (SAEs) related to the device or procedure. SAEs included events resulting in death, life-threatening conditions, hospitalization, disability, or incapacity. All adverse events (AEs) were recorded and evaluated by the Principal Investigator for severity, relationship to the procedure, and qualification as an SAE or unanticipated adverse device event (UADE).

#### *Secondary outcomes*:

##### • Crown fit:

Evaluated clinically based on the marginal, occlusal, and interproximal contacts and rated as 1-did not fit, 2-partial fit requiring significant modification, 3-neither a good nor bad fit, 4-good fit with requiring minor modification, 5-perfect fit, at the investigators’ sole discretion.

##### • Tooth preparation accuracy was evaluated using two methods:

- Shape of the Preparation: The prepared tooth scan was overlaid onto the planned tooth shape using a best-fit alignment based solely on the prepared surfaces.
- Location and Shape of the Preparation: The prepared tooth scan was aligned to the planned shape using best-fit alignment of the adjacent tooth occlusal surfaces, thereby assessing both the preparation’s location and shape.

##### • Patient-reported outcomes:

Patients scored the tooth, gingiva, jaw and head discomfort experienced during the procedure using the Wong-Baker Pain Rating Scale. Comfort during the procedure was rated by the patients on a scale as relaxed, a little uneasy, tense, anxious, or very anxious.

### Statistical analysis

As an early feasibility study, no formal sample size calculation was performed. Following the US FDA’s guidance for Early Feasibility Studies 5 to 10 participants were included. Data were analyzed descriptively without hypothesis testing. For continuous variables meeting normality, the mean, standard deviation, minimum, maximum, and coefficient of variation were calculated. For non-normal distributions, the median, interquartile range, and percentile distributions were reported. Analyses were performed using Minitab Statistical Software (Minitab LLC., State College, Pennsylvania, USA).

## Figures and Tables

**Table 1:** Preparation accuracy for the target teeth and evaluation of adjacent tooth damage.

Subject ID	RMS plan vs actual: target tooth goal < 120 μm	RMS plan vs actual: adjacent teeth goal < 200 μm	RMS plan vs actual: adjacent tooth damage goal < 200 μm, maximum single deviation 400 μm	Iatrogenic Tooth Damage (Qualitative Assessment) goal: no damage visible with unaided eye
001	38.3 μm	81.5 μm	66.7 μm (distal) 95.3 μm (mesial)	No damage visible with unaided eye
002	45.8 μm	104.4 μm	56 μm (distal) 91.7μm (mesial)	No damage visible with unaided eye
003	45.1 μm	109.7 μm	101.2 μm (distal) 0 (mesial)	No damage visible with unaided eye
004	44.3 μm	96.4 μm	0	No damage visible with unaided eye
005	32.8 μm	68.5 μm	123.4 μm	No damage visible with unaided eye
006	Withdrawn from study el Step-4, Tooth Fixation			
007	28.6 μm	90.3 μm	149.4 μm	Minor damage visible as expeded pet preappnoved tooth preparation plan

## Data Availability

The datasets generated during and/or analyzed during the current study are available from the corresponding author on reasonable request.
